# A Plasma Biomarker Panel of Four MicroRNAs for the Diagnosis of Prostate Cancer

**DOI:** 10.1038/s41598-018-24424-w

**Published:** 2018-04-27

**Authors:** Farhana Matin, Varinder Jeet, Leire Moya, Luke A. Selth, Suzanne Chambers, Australian Prostate Cancer BioResource, T. Yeadon, P. Saunders, A. Eckert, P. Heathcote, G. Wood, G. Malone, H. Samaratunga, A. Collins, M. Turner, K. Kerr, Judith A. Clements, Jyotsna Batra

**Affiliations:** 1 Australian Prostate Cancer Research Centre- Queensland, Translational Research Institute, Brisbane, QLD 4102 Australia; 2 Cancer Program, School of Biomedical Sciences, Institute of Health and Biomedical Innovation, Queensland University of Technology, Brisbane, QLD 4102 Australia; 3 Dame Roma Mitchell Cancer Research Laboratories, School of Medicine, Faculty of Health and Medical Sciences, Adelaide Medical School, Adelaide, SA 5000 Australia; 4 Menzies Health Institute Queensland, Griffith University, Gold Coast, QLD 4222 Australia; 5 Brisbane Urology Clinic, Brisbane, QLD 4000 Australia; 6 Aquesta Uropathology, Brisbane, QLD 4066 Australia; 7 Sullivan and Nicolaides Pathology, Brisbane, QLD 4006 Australia

## Abstract

Prostate cancer is diagnosed in over 1 million men every year globally, yet current diagnostic modalities are inadequate for identification of significant cancer and more reliable early diagnostic biomarkers are necessary for improved clinical management of prostate cancer patients. MicroRNAs (miRNAs) modulate important cellular processes/pathways contributing to cancer and are stably present in body fluids. In this study we profiled 372 cancer-associated miRNAs in plasma collected before (~60% patients) and after/during commencement of treatment (~40% patients), from age-matched prostate cancer patients and healthy controls, and observed elevated levels of 4 miRNAs - miR-4289, miR-326, miR-152-3p and miR-98-5p, which were validated in an independent cohort. The miRNA panel was able to differentiate between prostate cancer patients and controls (AUC = 0.88). Analysis of published miRNA transcriptomic data from clinical samples demonstrated low expression of miR-152-3p in tumour compared to adjacent non-malignant tissues. Overexpression of miR-152-3p increased proliferation and migration of prostate cancer cells, suggesting a role for this miRNA in prostate cancer pathogenesis, a concept that was supported by pathway analysis of predicted miR-152-3p target genes. In summary, a four miRNA panel, including miR-152-3p which likely targets genes with key roles in prostate cancer pathogenesis, has the potential to improve early prostate cancer diagnosis.

## Introduction

Prostate cancer is one of the most commonly diagnosed cancers in men worldwide with a forecast of increased incidence over the following years based on current trends^1^. Measuring the levels of prostate specific antigen (PSA; also known as human kallikrein-3) in serum is the standard first line test to indicate risk of prostate cancer and the most widely used biomarker for prostate cancer diagnosis. The advent of the PSA test in the late 1990s as a tool for prostate cancer diagnosis has enormously benefited prostate cancer patients for more than two decades^2,3^. However, inadequacies continue to exist over its suitability for use as a diagnostic tool for early detection of prostate cancer due to lack of specificity and high rates of over-diagnosis and over-treatment associated with PSA testing^4,5^. Therefore, there is an urgent clinical need for better diagnostic tools for prostate cancer.

Although prostate biopsy is the gold standard prostate cancer diagnostic tool, it is bound by several limitations^6^. Most prostate biopsies are routinely performed taking 12 cores under transrectal ultrasound guidance^6^, with an increase in the core number found to increase cancer detection rate by only 1.06 fold^7^. However, the major drawback lies in the possibility of generating false negatives, as the samples are often taken randomly due to the unknown location of the tumour, and patients may require repeated biopsies under MRI guidance or in combination with ultrasound for better sensitivity^6^. Over recent years, considerable research has led to the development of several molecular and genetic assays that have provided a prospective direction for the development of prostate cancer biomarkers^5^. Such assays utilise body fluid- or tissue-based biomarkers for the diagnosis, prognosis and risk stratification of prostate cancer^5^. Despite recent advances, it is still necessary to understand the role of these tests in the overall management of prostate cancer patients and the search for potential new biomarkers continues.

MicroRNAs (miRNAs) are a class of endogenous non-coding RNA molecules that play a pivotal role in gene regulation by binding to complementary target messenger RNAs (mRNAs), resulting in target mRNA degradation or translational blockade^8^. With the discovery that miRNAs exist in a stable form in clinical specimens such as plasma and serum^9^, urine^10^ and other body fluids^11^, miRNAs hold great promise as useful biomarkers. More recently, a number of studies have investigated the diagnostic, prognostic and risk stratification abilities of miRNAs in various diseases^12–14^, including prostate cancer^15–19^, which is summarised in our recently published review^20^.

In the present study, we investigated the differential expression of cancer-associated miRNAs in plasma samples collected from age-matched prostate cancer patients and healthy control individuals using miRNA PCR array profiling followed by quantitative real-time PCR (qRT-PCR). We evaluated the ability of the identified miRNAs to diagnose prostate cancer in an independent sample-set for validation of our results. Furthermore, we assessed the prostatic expression of these miRNAs in tumour versus adjacent non-malignant tissues in The Cancer Genome Atlas (TCGA) dataset, and explored the functional role of one putative miRNA biomarker in prostate cancer cell proliferation and migration using cell-based functional assays. Our study reveals disease-relevant miRNAs with potential to improve the diagnosis of prostate cancer.

## Results

### miRNA screening from pooled plasma samples

**Figure 1:**
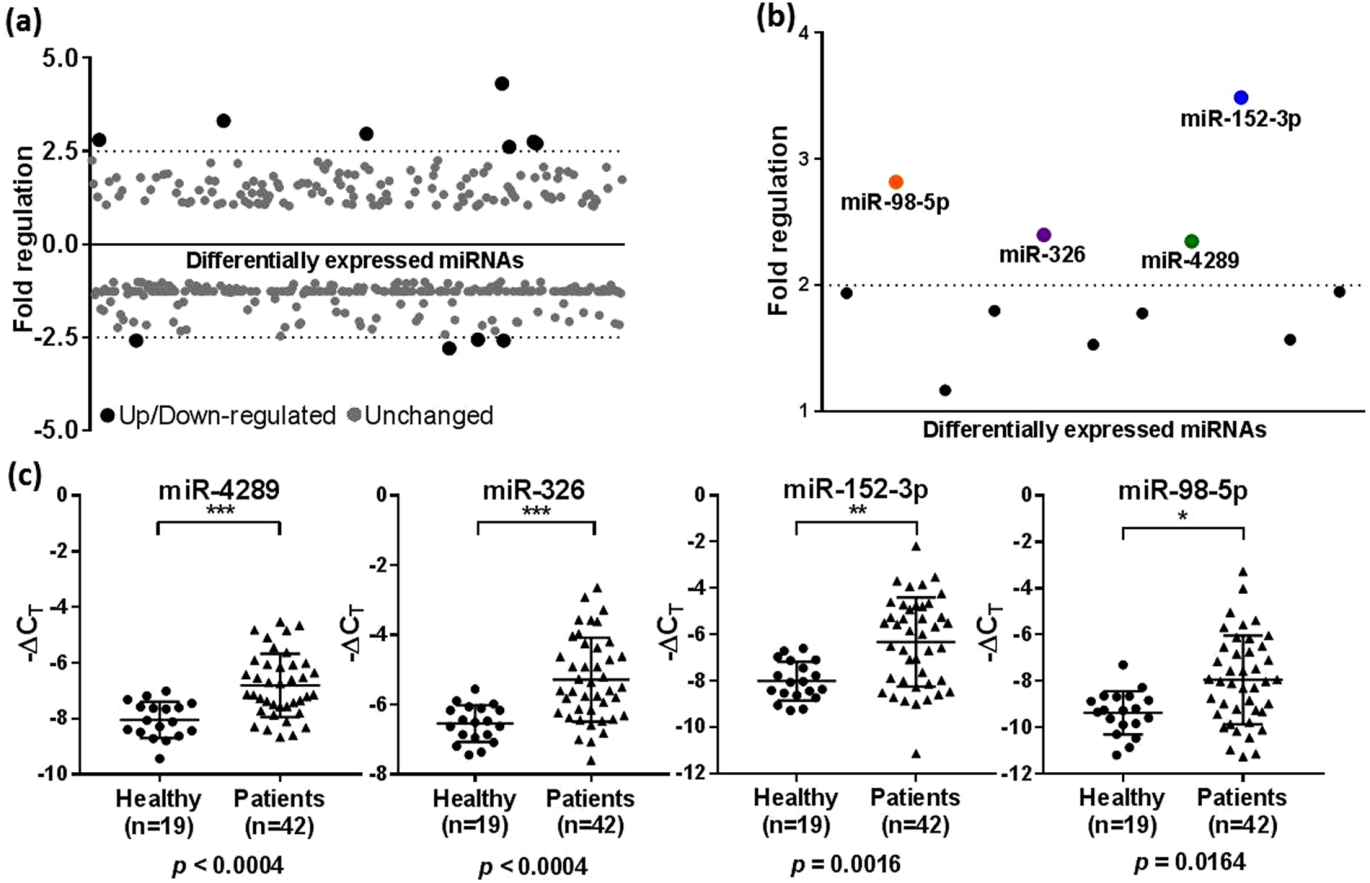
Screening of plasma miRNA markers in pooled patient and control samples and validation in the discovery cohort. **(a)** Scatter plot of 372 cancer-associated miRNAs in a screening cohort of pooled plasma samples from prostate cancer patients and healthy controls screened using a miScript miRNA PCR array. miRNAs found to be differentially expressed between the patient and control groups are shown in black, and unchanged miRNAs are shown in grey. A fold regulation cut-off of 2.5 was selected for the analysis. **(b)** Scatter plot showing the 11 differentially regulated miRNAs re-analysed by qRT-PCR in patient samples in the discovery cohort (N = 61). The mean fold regulation of each miRNA across the patient and control samples was taken into account and those that were below the 2 fold regulation cut-off were excluded from further analysis. The selected miRNAs are shown in colour. **(c)** Relative levels of miR-4289, miR-326, miR-152-3p and miR-98-5p analysed by qRT-PCR as in (**b**) in patients vs healthy controls. Statistically significant differences were assessed using a Mann-Whitney U test; p values are shown after Bonferroni correction for multiple testing. Each data point represents a plasma sample, the horizontal middle line in each data set represents the mean, and the limits of the vertical lines represent the standard deviation.

Differential expression of miRNAs in pooled plasma samples from prostate cancer patients and healthy controls was measured by qRT-PCR using miScript miRNA PCR arrays, which contained pre-loaded miScript Primer Assays for 372 cancer-associated miRNAs plus 12 internal controls. Most of the miRNAs were detectable in the plasma samples as indicated by their C_T_ values (Supplementary Table S1), and only a small percentage of miRNAs were undetected in the patient group and control group, indicating the high sensitivity of the method (Supplementary Figure S1). Through extensive data analysis of our miRNA PCR array data, we shortlisted 11 deregulated miRNAs, of which 7 were up-regulated and 4 were down-regulated in patient samples when compared to healthy controls (Fig. 1a; Supplementary Table S2).

### Expression of selected plasma miRNAs in discovery cohort

To validate the findings from the screening study, we quantitated the 11 candidate miRNAs by qRT-PCR in individual plasma samples in the discovery cohort (N = 61) consisting of plasma samples collected from age-matched patients (N = 42) and controls (N = 19). The clinical characteristics of the participants are summarised in Supplementary Table S3. The 7 up-regulated miRNAs in the screening study were differentially expressed in the same direction. However, the 4 down-regulated miRNAs were up-regulated in individual patient samples; due to this discrepancy between the analyses, these candidates were discarded. Of the remaining miRNAs, 4 miRNAs - miR-4289, miR-326, miR-152-3p and miR-98-5p, had a mean fold-regulation value of >2 and were all significantly altered compared to controls as determined by the Unpaired Mann-Whitney U test followed by multiple testing using Bonferroni correction, p ≤ 0.05 (Fig. 1b/c).

### Validation of plasma miR-4289, miR-326, miR-152-3p and miR-98-5p

To further validate our results from the discovery cohort, we assessed an independent set of patient and control samples (N = 58). This validation cohort comprised of 40 patients and 18 controls. 19 out of 40 patients (i.e. 47.5%) in the validation cohort had a Gleason score of 7, in contrast to patients in the discovery cohort where the Gleason score was 9 in 18 out of 42 patients (i.e. 42.9%). 9 out of 42 patients (i.e. 21.4%) in the discovery cohort had a Gleason score of 7, and 7 out of 40 patients (i.e. 17.5%) in the validation cohort had a Gleason score of 9. The mean age of patients in the two cohorts was similar (i.e. 65.4 ± 1.3 s.d. years and 63.3 ± 1.1 s.d. years) and the controls were age-matched accordingly (Supplementary Table S3).

**Figure 2:**
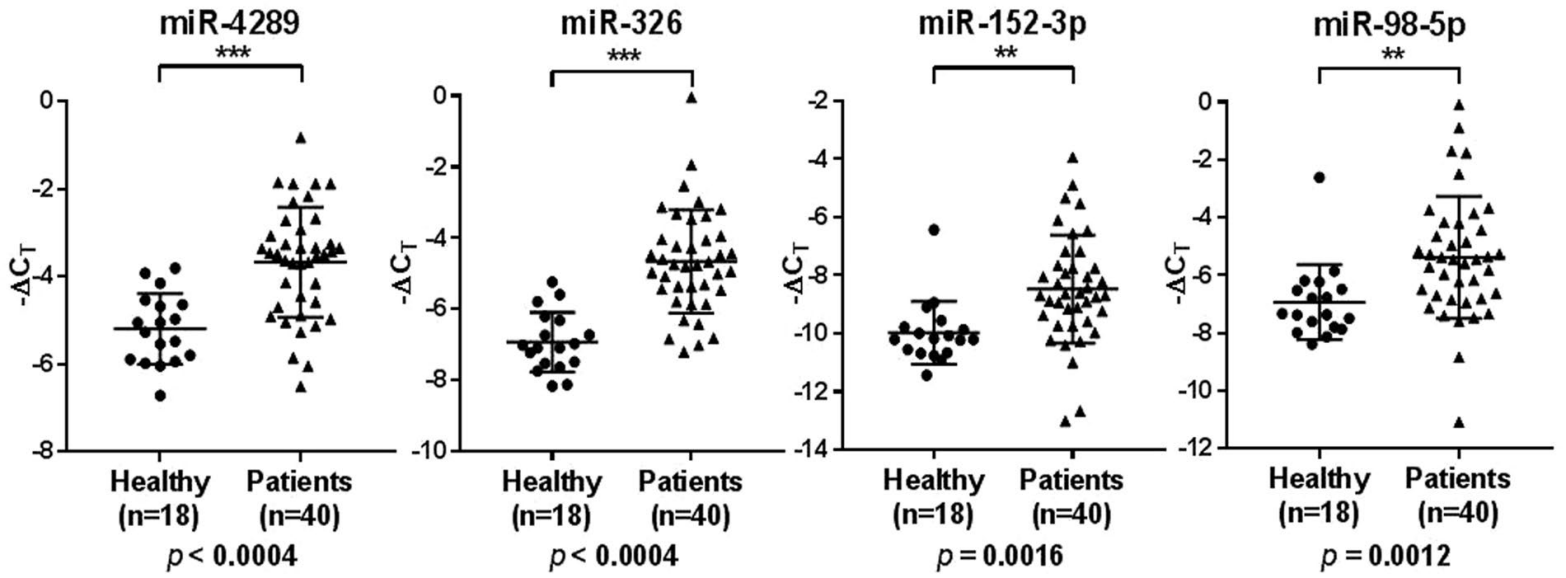
Relative expression of miR-4289, miR-326, miR-152-3p and miR-98-5p in the validation cohort (N = 58). Statistically significant differences in miRNA expression levels between the patients and control groups were assessed using a Mann-Whitney U test; p values are shown after Bonferroni correction for multiple testing. Each data point represents a plasma sample, the horizontal middle line in each data set represents the mean, and the limits of the vertical lines represent the standard deviation.

Differential expression analysis in the validation cohort confirmed that the levels of miR-4289, miR-326, miR-152-3p and miR-98-5p were significantly altered in patients compared to controls using Unpaired Mann-Whitney U test followed by multiple testing using Bonferroni correction, p ≤ 0.05 (Fig. 2).

### Diagnostic miRNA signature for prostate cancer

Given the association of miRNA expression with prostate cancer, we performed univariate logistic regression analysis on the discovery (N = 61) and validation (N = 58) cohorts for each of the four miRNAs (Table 1). The regression coefficient (B) indicates an estimated increase in the odds of the outcome (i.e. prostate cancer) with increase in the value of the exposure (i.e. plasma miRNA level). The 95% confidence interval (CI) was used as a measure of precision of the regression coefficient and to determine the presence of statistical significance which was confirmed by the *p*-value. miR-4289, miR-326 and miR-152-3p were found to be significantly associated with disease occurrence in the discovery cohort, except for miR-98-5p which did not reach statistical significance in this cohort (Table 1). All four miRNAs were significant in the validation cohort (Table 1). Adjustments were made for each patient and control sample for varying starting concentration of RNA using off-set correction during binary logistic regression analysis.

**Table 1:** Univariate logistic regression analysis on the discovery (N = 61) and validation (N = 58) cohorts for each of the four miRNAs.

miRNA	Discovery cohort (N = 61)			Validation cohort (N = 58)		
	Regression coefficient (B)	95% CI	*p*-value	Regression coefficient (B)	95% CI	*p*-value
miR-4289	0.83	0.50–1.16	3.11E − 06	5.71	4.18–7.24	8.80E − 13
miR-326	6.18	4.52–7.83	9.15E − 13	10.42	8.09–12.76	0.00E + 00
miR-152-3p	1.89	0.99–2.79	1.40E − 04	3.38	2.27–4.49	8.94E − 09
miR-98-5p	1.75	0.36–3.14	0.056	1.80	1.20–2.40	1.47E − 08

The regression coefficient (B) was used to determine the association of the outcome with an increase in plasma miRNA expression. The 95% confidence interval (CI) was used as a measure of precision of the regression coefficient and to determine the presence of statistical significance as confirmed by the *p* value. miR-4289 (p = 3.11E-06, 95% CI = 0.50–1.16, std. error = 0.17), miR-326 (p = 9.15E-13, 95% CI = 4.52–7.83, std. error = 0.84) and miR-152-3p (p = 1.40E-04, 95% CI = 0.99–2.79, std. error = 0.46) were found to be significantly associated with disease in the discovery cohort, except for miR-98–5p (p = 0.056, 95% CI = 0.36–3.14, std. error = 0.71) which did not reach statistical significance in this cohort. miR-4289 (p = 8.80E-13, 95% CI = 4.18–7.24, std. error = 0.78), miR-326 (p = 0.00E + 00, 95% CI = 8.09–12.76, std. error = 1.20) and miR-152-3p (p = 8.94E-09, 95% CI = 2.27–4.49, std. error = 0.56) and miR-98-5p (p = 1.47E-08, 95% CI = 1.20–2.40, std. error = 0.31) were significant predictors of disease in the validation cohort. Adjustments were made for each patient and control sample for varying starting concentration of RNA using off-set correction during binary logistic regression analysis.

**Figure 3:**
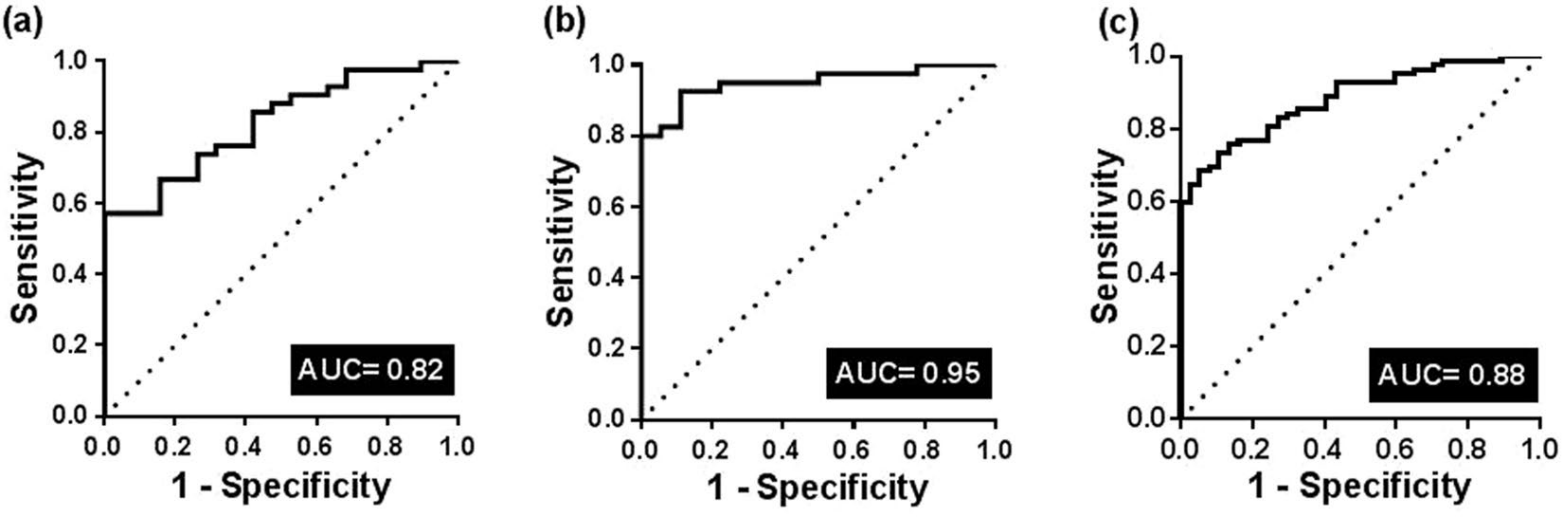
ROC curve analysis in the discovery, validation and combined cohorts comparing the ability of the miRNA signature to identify prostate cancer patients. (**a**) A combined measure of the sensitivity and specificity of the miRNA signature in the discovery cohort (N = 61) is represented by the Area under the curve AUC = 0.82 (p < 0.0001, 95% CI = 0.72–0.93). (**b**) A combined measure of the sensitivity and specificity of the miRNA signature in the validation cohort (N = 58) is represented by AUC = 0.95 (p < 0.0001, 95% CI = 0.89–1.00). (**c**) A combined measure of the sensitivity and specificity of the miRNA signature in a combined cohort (N = 119) is represented by AUC = 0.88 (p < 0.0001, 95% CI = 0.82–0.94). The diagonal reference line reflects the performance of the diagnostic test i.e. whether a test yields the positive or negative results by chance or due to a relation with the true disease status.

We further evaluated, using multivariate binary logistic regression and ROC analyses, the diagnostic capacity of the individual and combination of miR-4289, miR-326, miR-152-3p and miR-98-5p in the discovery and validation cohorts as well as in both cohorts combined. ROC analysis for each miRNA in the panel was also performed for the discovery, validation and combined cohorts to allow comparison between the AUC of each miRNA and the AUC of all four miRNAs together as a measure of diagnostic accuracy (Supplementary Figure S2). The AUC for individual miRNAs ranged from 0.69–0.85 for miR-4289, 0.82–0.91 for miR-326, 0.72–0.80 for miR-152-3p and 0.70–0.79 for miR-98-5p respectively (Supplementary Figure S2). The combined predictive probability of all four miRNAs produced an Area under the curve (AUC) of 0.82 (p < 0.0001, 95% CI = 0.72–0.93) in the discovery cohort (Fig. 3a), AUC = 0.95 (p < 0.0001, 95% CI = 0.90–1.00) in the validation cohort (Fig. 3b) and AUC = 0.88 (p < 0.0001, 95% CI = 0.82–0.94) in the combined cohort (Fig. 3c) for the discrimination of prostate cancer patients from healthy controls. Despite some differences in the Gleason score between the two cohorts, the diagnostic accuracy of the miRNA signature was similar in the discovery, validation and combined cohorts.

### miRNA expression in transcriptomic data from clinical samples

**Figure 4:**
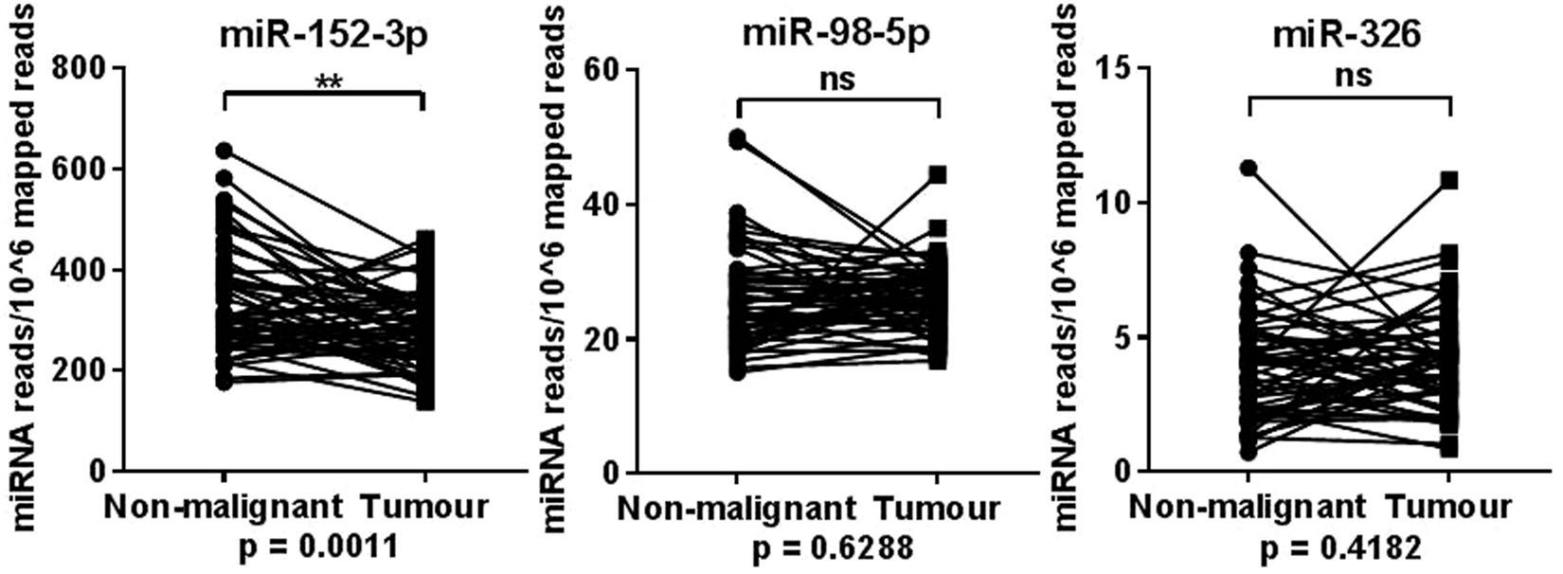
Analysis of published miRNA transcriptomic data from clinical samples. TCGA data expression analysis of miR-152-3p, miR-98-5p and miR-326 in 52 tumour and adjacent non-malignant prostate tissues. The expression of miR-152-3p was significantly lower (p = 0.0011) in tumour compared to adjacent non-malignant prostate tissues, while the expression of miR-98-5p and miR-326 did not reach statistical significance (p = 0.6288 and p = 0.4182). TCGA data was not available for miR-4289. The differences between the paired samples were assessed using a Wilcoxon test.

Since there may be differences between the expression of miRNAs in the circulation and in tumour tissues, we next determined whether the plasma levels of the identified miRNAs reflected similar changes in tumour tissues. Analysis of published miRNA transcriptomic data from clinical samples in the TCGA dataset demonstrated low expression of miR-152-3p in tumour compared to adjacent non-malignant tissues (*p* = 0.001) (Wilcoxon test, p ≤ 0.05) (Fig. 4) (Supplementary Table S4). No substantial expression changes were observed for miR-98-5p and miR-326 (Supplementary Table S4), and information on miR-4289 expression was unavailable in the processed TCGA dataset.

### miR-152-3p increases proliferation and migration of prostate cancer cells

**Figure 5:**
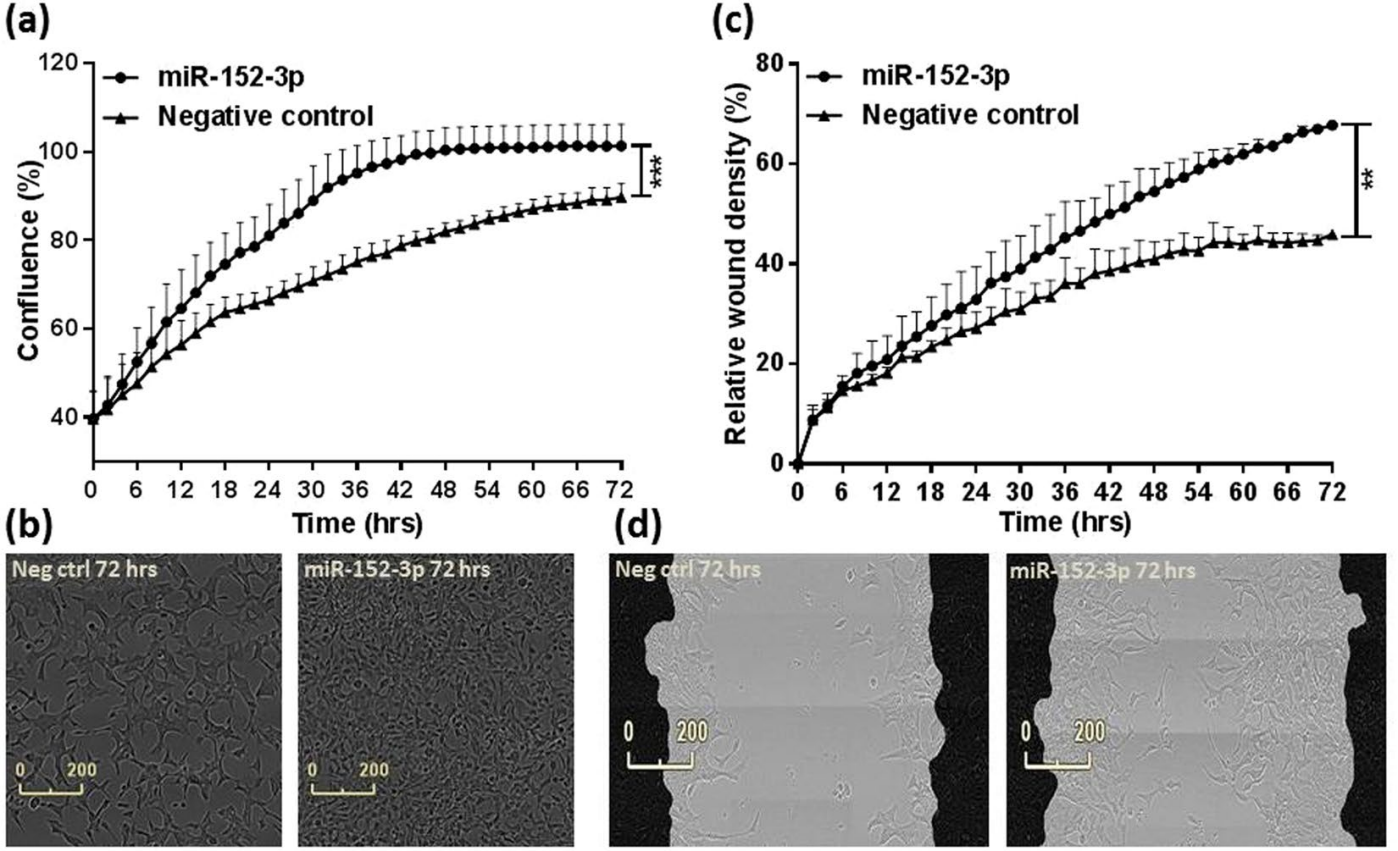
miR-152-3p mediates cell proliferation and migration in LNCaP cells. **(a)** Overexpression of miR-152-3p using miRNA mimics in LNCaP cells increased their proliferative capacity (p = 0.0002) measured as an increase in percentage confluence by the IncuCyte live-cell imaging system. **(b**) Overexpression of miR-152-3p also increased migration in LNCaP cells (p = 0.0084) measured as an increase in percentage relative wound density. **(c)** An increase in proliferation was accompanied by a change in morphology in miR-152-3p treated LNCaP cells when compared to non-targeting negative control treated cells. **(d)** LNCaP cells were grown to form a confluent monolayer before scratches were made and wound healing was measured by the IncuCyte system. Both the functional assays were performed for a period of 72 hours and data was collected at every 2 hour time point throughout the experiments. The differences between the miR-152-3p and negative control treated cells were assessed using a Mann-Whitney U test, N = 3.

We further determined the role of miR-152-3p in proliferation and migration of prostate cancer cells. Cell line expression analysis of a panel of prostate cell lines showed that miR-152-3p is lowly expressed in the LNCaP and PC3 prostate cancer cell lines compared to the benign prostatic hyperplasia (BPH) epithelial cell line with highest expression in RWPE-2 cells derived from the normal RWPE-1 cell line (Supplementary Figure S3). Our results suggest a significant (*p* = 0.0002) increase in the proliferative potential of LNCaP cells upon overexpression of miR-152-3p using miRNA mimics (Fig. 5a). We observed a substantial increase in cell confluence and change in cell morphology in miR-152-3p mimic treated cells when compared to non-targeting negative control treated cells over a period of 72 hours (Fig. 5b; Supplementary Videos 1–2). Similarly, the rate of migration as indicated by wound closure was significantly (*p* = 0.0084) pronounced with miR-152-3p treatment when compared to the negative control (Fig. 5c,d; Supplementary Videos 3–4). We did not observe any significant effects on the proliferation of PC3 cells with miR-152-3p treatment over a period of 72 hours (Supplementary Figure S4).

### Potential target genes and pathways altered by the identified miRNAs

Identifying the target genes of the putative miRNA biomarkers is important in understanding their functions. *In silico* pathway analysis using the miRNet web-based platform generated target genes and candidate pathways for the miRNAs (Supplementary Figure S5; Supplementary Tables S5 and S6). The miRNet predicted targets are determined experimentally as part of larger experiments such as microarray, RNA-seq, qPCR and PAR-CLIP, an immunoprecipitation method for identifying the binding sites of cellular RNA-binding proteins (RBPs) and microRNA-containing ribonucleoprotein complexes (miRNPs) and each identified target gene is supported by the experiment name and PubMed literature^21^. Based on the analysis of miRNA expression in the TCGA dataset and functional assays, miR-152-3p targets were used as an input for Ingenuity Pathway Analysis (IPA).

**Figure 6:**
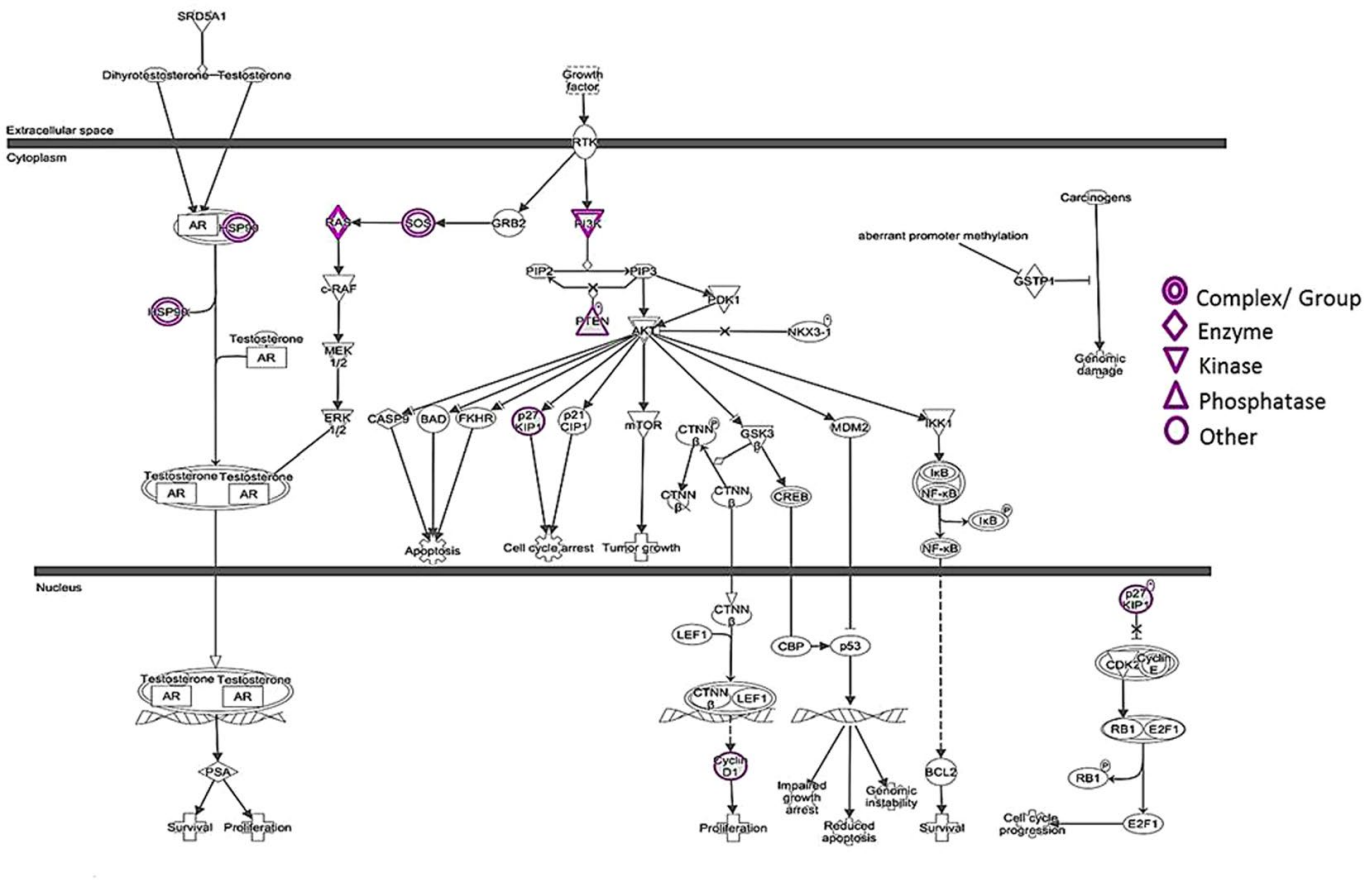
Ingenuity pathway analysis (IPA) of miR-152-3p-target interactions in prostate cancer signalling. Prostate cancer signalling was among the top ten canonical pathways consisting of eight deregulated miR-152-3p target genes (highlighted in purple) i.e. FGFR3, IRS1, SOS2, HSP90AA1, KRAS, CDKN1B, CCND1 and PTEN where FGFR3 and IRS1 are closely related to PI3K and hence shown as a group. Some of the targets for e.g. HSP90, KRAS and SOS exist as a complex or group of genes. The p value was represented as −log *p* = 5.57 for the analysis.

IPA generated 275 canonical pathways for the 131 targets of miR-152-3p (Supplementary Table S7). Prostate cancer signalling was among the top ten canonical pathways consisting of 8 deregulated target genes (highlighted in purple) i.e. FGFR3, IRS1, SOS2, HSP90AA1, KRAS, CDKN1B, CCND1 and PTEN of miR-152-3p (−log *p* = 5.75) (Fig. 6) where FGFR3 and IRS1 are closely related to PI3K and hence shown as a group. Analysis of the molecular function of these genes confirmed their involvement in cellular processes such as cell cycle, cell morphology, cell growth and proliferation and cell movement (Supplementary Table S8). The analysis also generated a list of upstream regulators of the 8 target genes (Supplementary Table S9). Of the 8 target genes of miR-152-3p, six overlapped with GSEA where prostate cancer was found to be the second most important pathway among the top 20 KEGG pathways (Supplementary Figure S6). Furthermore, Oncomine data analysis of these 8 genes showed significant upregulation of CCND1 (*p* = 9.88E-6), FGFR3 (*p* = 0.002) and PTEN (*p* = 0.022) in prostate carcinoma (N = 65) compared to normal (N = 23) tissues in the Yu Prostate Cancer Dataset (Supplementary Figure S7a,b), however, five i.e. HSP90AA1 (*p* = 7.92E-14), CDKN1B (*p* = 1.67E-12), CCND1 (*p* = 4.50E-9), PTEN (*p* = 5.70E-6) and KRAS (*p* = 0.021) out of 8 target genes were significantly upregulated during prostate cancer metastasis (Supplementary Figure S7c).

### miR-152-3p modulates IRS1, HSP90AA1 and KRAS expression at the mRNA level

qRT-PCR analyses of six predicted target genes (CCND1, IRS1, PTEN, HSP90AA1, KRAS and CDKN1B) of miR-152-3p after treatment with miR-152-3p and non-targeting negative control mimics in LNCaP cells for 72 hours showed a significant reduction in IRS1 (p = 0.0014) and HSP90AA1 (p = 0.0007) mRNA, and increased expression of KRAS (p < 0.0001) at the mRNA level suggesting these three genes as direct/indirect targets of miR-152-3p (Supplementary Figure S8).

## Discussion

Circulating miRNAs in body fluids may serve as clinically important biomarkers of various malignancies, including prostate cancer. In the present study, we identified an association of elevated plasma levels of four miRNAs i.e. miR-4289, miR-326, miR-98-5p and miR-152-3p in prostate cancer patients. Initially, we analysed the plasma miRNAome in age-matched prostate cancer patients and healthy controls using a miRNA PCR array approach. We selected sample pooling for initial screening of miRNAs because it minimised cost, reduced analytical run times and compensated for limited amounts of plasma samples enabling us to profile a large number of miRNAs in patient and control samples in a cost effective way, and has been employed in previous studies^22,23^. Many biological experiments have relied on pooling of biological specimens and the efficiency of this method has also been statistically investigated previously^24,25^. Although sample pooling has its own advantages, it is not the optimum approach because while mitigating the loss of information, it does not take into consideration biological variation within individual samples. This may lead to the difference observed between miRNA expression in pooled and individual samples. Therefore, subsequent qRT-PCR in single samples from the discovery and validation cohorts was performed for validation of results and only those miRNAs that were deregulated in the same direction in both pooled and individual samples were selected for further analysis by applying a stringent cut-off. The majority of previously conducted studies have used a higher C_T_ cut-off to identify multiple miRNAs, however, we have applied a strict cut-off to our analysis as our goal was to determine the most obvious candidates for rapid identification along the line of clinical translation. Our identified miRNA signature generated ROC curves with AUC values better than the previously reported AUC value for PSA^26,27^. Similarly, other groups have assessed the diagnostic performance of plasma or serum miRNAs in patients with localised or metastatic prostate cancer, BPH and healthy controls, and in most instances the specificity and sensitivity of the miRNA biomarkers have outperformed the accuracy of the PSA test^23,28,29^. Although early detection and management of prostate cancer is a complex ongoing issue, there is insufficient evidence to support the benefits of population-based screening for prostate cancer in Australia and in the United States using the PSA test^30–32^. The Cancer Council Australia and U.S. Preventative Services Task Force recommends against the PSA test, partly due to the over-diagnosis and over-treatment associated with it^33,34^. PSA screening in healthy men is uncommon in Australia and thus, we did not have the PSA values for our control group which limited our ability to perform the miRNA vs PSA comparisons in our study. A pertinent limitation of our study is that some (45% and 42%, respectively) patient samples in cohort-1 and 2 were collected after or during primary treatment. Although miRNA levels are affected by the treatment regimen of patients, we have tried to mitigate this effect by keeping the timing of sample collection consistent between the discovery and validation cohorts, using appropriate statistical methods and relying on the stability of miRNAs in body fluids.

Our study has implicated miR-4289 as a prostate cancer diagnostic biomarker for the first time. To date, only two studies investigating the role of miR-4289 in congenital obstructive nephropathy as an exosomal biomarker^35^, and Middle East Respiratory Syndrome (MERS) caused by a human coronavirus^36^ have been published. Hasan *et al*. proposed the use of host miR-4289 as an antiviral therapy for MERS^36^ which may provide further clues to explore its therapeutic potential in prostate cancer.

miR-326 has been identified in a recent study by Kristensen *et al*. as a member of a new 3-miRNA classifier consisting of miR-185-5p, miR-221-3p and miR-326 that predicted biochemical recurrence (BCR) after radical prostatectomy in prostate cancer patients^37^. The study was one of the largest miRNA expression profiling studies in tumour tissue samples from 126 patients in the discovery cohort and 209 patients in the validation cohorts^37^. However, an increased level of plasma miR-326 has not been previously reported as an early diagnostic marker for prostate cancer. miR-326, as a member of a five miRNA signature has been correlated with the survival of patients with hepatocellular carcinoma, and identified as one of three miRNAs applicable for the diagnosis of these patients in a more recent study^38^.

Recent studies have indicated the importance of miR-98 in colon^39^, lung^40^, liver^41^ and prostate^42^ cancer. Meta-analysis of six miRNA datasets revealed up-regulation of 15 miRNAs, miR-98 being one of them, in recurrent compared to non-recurrent prostate samples suggesting the predictive ability of miR-98 along with other miRNAs^42^. To the best of our knowledge, we are the first to demonstrate plasma miR-98-5p as a candidate for early diagnosis of prostate cancer.

Finally, miR-152 has previously been shown to be substantially decreased in higher Gleason grade prostate cancer tissues and its low expression was correlated with advanced pathological stages^43,44^. The expression pattern is in line with our findings in the TCGA dataset where miR-152 is lowly expressed in tumour compared with adjacent non-malignant tissues. A possible mechanism for the decreased expression is likely to be methylation of the miR-152 host gene promoter which prevents transcription of this miRNA in tumour tissues possibly due to its tumour suppressive nature^45^. Another mechanism could be its release into the circulation to enable tumour growth at metastatic sites which may explain our results obtained through functional assays using miR-152-3p mimics. Elevated levels of plasma miR-152 have been proposed as a diagnostic biomarker for other cancers such as lung, colorectal and breast cancer^46^, and our findings suggest its potential to detect prostate cancer either alone or in combination with the other three identified miRNAs.

Although higher levels of these miRNAs may be indicative of prostate cancer pathogenesis, their mechanism of action is difficult to determine. Studies have reported that circulating miRNAs are involved in cell to cell communication and can be taken up by recipient cells to exert functional effects such as proliferation, invasion and angiogenesis^47^. For example, tumour exosomal miRNAs were shown to promote metastasis in other sites of the body by modulation of stromal cells in distant organs^48^. We did not observe any significant effect of miR-152-3p overexpression on the proliferative ability of PC3 prostate cancer cells as previously suggested by Zhu *et al*.^43^. In contrast, we found that overexpression of miR-152-3p significantly increased proliferation and migration in LNCaP prostate cancer cells which suggests that regulation by the androgen receptor (AR) may account for the observed differences. This is contradictory to the findings from another study by Theodore *et al*. where low expression of miR-152 has been correlated with an increase in prostate cancer metastasis^44^. However, this may provide a clue to the metastatic role of this miRNA at a distant site other than its site of production possibly via exosomal escape as miRNAs are known to play a role in metastasis^49,50^ and miR-152-3p has been previously reported as an exosomal miRNA^51,52^. It is also believed that miRNA secretion is a selective process and therefore, circulatory levels are not a true reflection of intracellular levels^53^. On the contrary, some miRNAs are released by tumour cells as Argonaute (AGO) bound complexes^54^ or from dead cells in apoptotic bodies^55^, thus indicating the tumour as one of their sources.

Identification of miRNA-target genes and pathways to understand the molecular basis of prostate cancer pathogenesis is a major challenge, as there are several risk factors and numerous pathways that drive cancer. Of the four miRNAs, miR-152-3p targets were selected for further analysis due to the significant differential expression of this miRNA in the TCGA dataset, and our findings from the functional assays. In depth analysis of miR-152-3p targets generated 10 target genes through IPA and GSEA in the prostate cancer signalling pathway with six common targets between the two analyses. The six genes were found to be important modulators of cellular processes involved in cancer progression.

CCND1 is a well-recognised oncogene involved in the direct phosphorylation of the retinoblastoma (Rb) protein and promoting cell cycle transition from the G1 to S phase^56^. CCND1 is over-expressed in a considerable portion of human malignancies such as breast and prostate cancer^57,58^ and it has been shown as a miRNA target in breast cancer cells and primary prostatic epithelial cells^56^. Similarly, recent studies by Lynch *et al*. and Chakravarthi *et al*. demonstrated an inverse correlation between CDKN1B and miRNAs in prostate cancer cell lines and clinical prostatectomy specimens^59,60^. However, we did not observe any change in CCND1 and CDKN1B mRNA expression in LNCaP cells with miR-152-3p treatment. KRAS is a well-established oncogenic GTPase protein playing an important role in cell division, differentiation and apoptosis. miRNA targeting of the KRAS 3′UTR in a recent study induced cell apoptosis *in vitro* and exerted a tumour suppressive effect *in vivo* in xenograft mice models of colorectal cancer^61^. This suggests the importance of the miRNA-KRAS axis in prostate cancer where the miRNA has been found to play a tumour suppressive role^62^. On the contrary, our results suggest the upregulation of KRAS at the mRNA level with miR-152-3p treatment in LNCaP cells which may explain the increase in cell proliferation and migration observed during our functional studies. HSP90AA1 is a molecular chaperone that promotes regulation of specific proteins involved in signal transduction and cell cycle. A recent study by Wilson *et al*. indicated the importance of histone methylation of AR binding sites of transcriptional targets, such as HSP90AA1 in androgen signalling in prostate cancer progression^63^. This may result in a positive regulation of HSP90AA1 in metastasis as shown by our Oncomine data analysis, whereas the expression of HSP90AA1 possibly remains unchanged in localised cancer due to more stable complex formation between AR and HSP90AA1. We observed a decrease in HSP90AA1 mRNA in LNCaP cells after miRNA treatment suggesting it as a possible target of miR-152-3p which in turn may play a role in the androgen signalling pathway. SOS2 is a guanine nucleotide exchange factor involved in Ras activation which stimulates a series of signal cascades crucial for malignant transformation. A recent study by Alles *et al*. demonstrated that miRNA overexpression in the HEK-293T kidney cancer cell line lead to reduced levels of endogenous SOS2^64^. Therefore, targeting of SOS factors by miRNAs may potentially inactivate Ras signalling, however, many other factors are involved in signalling cascades resulting in cancer progression.

PTEN is a lipid and protein phosphatase capable of regulating the PI3K-AKT pathway and suppress tumour growth in many cancers. PTEN expression and function is regulated by various mechanisms, including regulation by miRNAs^65^, and its co-inactivation has been reported to increase the neuroendocrine phenotype, a hallmark of prostate cancer progression during anti-androgen therapy^66^. Targeting of PTEN by miR-152-3p may therefore, result in increased cell proliferation and migration as observed in our functional assays, however, we did not observe any changes at the PTEN mRNA level with miR-152-3p treatment. Contrary to the previously reported role of PTEN, we also found that PTEN was up-regulated in both prostate cancer vs normal, and metastasis vs primary prostate cancer. The differential expression of PTEN was extracted in particular from the Yu Prostate dataset^67^ in the Oncomine cancer microarray database^68^. This dataset was selected over other datasets because it had the largest number of prostate cancer (primary/metastasis) and normal samples for comparison of all the target genes of interest. Although PTEN is generally considered to be a tumour suppressor, it may act as a tumour promoter is some instances^69^. It is also widely known that in addition to gene expression, genomic instabilities, mutations and copy number variation in the PTEN gene are often associated with prostate cancer^70,71^, and it is estimated that up to 70 percent of primary prostate tumours have PTEN gene mutations^71^. The Yu Prostate dataset in the Oncomine cancer microarray database did not take these into account during data analysis and as a result, PTEN may be shown to be up-regulated. Additional data analysis using other datasets in Oncomine taking PTEN deletions and copy number variations into account revealed trends in the opposite direction.

Evidence suggests that the target genes discussed so far play a vital role in prostate cancer and their regulation by miRNAs may be crucial in prostate cancer pathogenesis. Some of these miR-152-3p targets are oncogenes while some are tumour suppressors and this suggests that miRNA target modulation is dependent on various aspects such as genetic alterations, transcriptional/ post-transcriptional regulation, post-translational modification, target stabilisation, indirect regulation as well as cell type. Moreover, validation of target genes and further functional studies are necessary to understand the mechanistic role of our identified miRNAs in cancer progression.

In conclusion, a major strength of our study is that it is one of the largest scale studies conducted on Australian men to determine the potential of miRNAs as diagnostic markers of prostate cancer. Although it may have been ideal to include similar numbers of patients and controls, several published studies where the control group is smaller in number compared to the patient group have been reported previously^20^. In addition, we have identified a novel plasma miRNA panel consisting of four miRNAs which have previously not been implicated for early diagnosis of prostate cancer. This was achieved by using higher than normal stringency to identify the most reliable candidates which is unique compared with many other studies. It was beyond the scope of our study to compare miRNA expression levels in BPH patients as analysed in several other studies as discussed in our review article^20^. Developments in the field of biomarker research are ongoing as stipulated by emerging miRNAs and current tests that have a specific role in prostate cancer diagnosis and treatment. However, a critical need for robustness and reproducible data to implement such biomarkers in clinical practice still remains. Undoubtedly, in the next few decades a substantial number of biomarkers will be available for clinical use in the overall management of prostate cancer.

## Materials and Methods

### Patients and blood collection

This is a retrospective study examining miRNAs as risk/protection factors in relation to a diagnostic outcome. Prostate cancer patients were recruited as part of the Prostate Cancer Supportive Care and Patient Outcomes Project (ProsCan), a randomised controlled trial of a psychological intervention for newly diagnosed prostate cancer patients^72,73^ for the discovery cohort (N = 42). The validation cohort of prostate cancer patients (N = 40) were recruited as part of the Australian Prostate Cancer BioResource (APCB) consisting of 3,100 patients including 350 men recruited via collaborations with urologists, 2,000 men from the QLD node of APCB, and 750 men recruited in collaboration with The Cancer Council Queensland^73^. Healthy male control participants for both the discovery and validation cohorts (N = 37) were recruited through the electoral roll as part of the Queensland Men’s Health Study (QLDMen), a cross-sectional population-based study consisting of 1,300 men including 450 age- and postal code- matched controls to complement participants in the ProsCan and APCB studies. The patient and control numbers were selected depending on the availability of samples from participants with similar clinical characteristics, as well as with reference to previous studies that we have discussed in our published review article^20^. The patient group in each cohort is further divided into metastatic (≤5 and >5 years survival) and non-metastatic patients. This subdivision ensured that each category of participants i.e. metastatic, non-metastatic and healthy in each cohort consisted of about 20 samples. Follow-up data for ~10 years was available for the patients including those who survived for less than 5 years after prostate cancer diagnosis. Details of age, PSA levels, clinical grade (Gleason scores), pathologic state (TNM stages) and family history have been collected to document clinical characteristics of the disease. ~19–22% of the patients and ~11–15% of the healthy controls in each cohort had a family history of prostate cancer. 52.4–62.5% patients and 84.2–88.9% controls in both the cohorts did not have any family history of prostate cancer.

Blood samples were collected within 1–4 months of diagnosis and within 1–2 months of treatment, which included radical prostatectomy, androgen deprivation therapy (ADT), brachytherapy and/or radiation therapy, from patients in the discovery cohort (N = 61). Similarly, for the validation cohort (N = 58) blood was collected within 1–4 months of diagnosis and 1–2 months of any treatment, or at the time of radical prostatectomy for most of the patients, and for some collection was done after 4–9 months of hormone deprivation. Although miRNA levels are affected by the treatment regimen of patients, we have tried to mitigate this effect by keeping the timing of sample collection consistent between the discovery and validation cohorts, using appropriate statistical methods and relying on the stability of miRNAs in body fluids^74^. Blood samples were collected in ethylene-di-amine-tetra-acetic acid (EDTA) containing tubes and processed within 1 hour of collection. The resultant plasma samples were stored at −80 °C until RNA extraction was performed, and transported on dry ice to the Institute of Health and Biomedical Innovation (IHBI) for RNA isolation. All clinical samples were obtained from patients and controls after their written informed consent. The study was performed in accordance with the institutional ethics approval- Approval numbers: 3629H (ProsCan), 1000001165 (APCB), and 0600000216 (QLDMen) from the Queensland University of Technology (QUT) and the Cancer Council Queensland (CCQ).

### Plasma RNA isolation and cDNA synthesis

**Figure 7:**
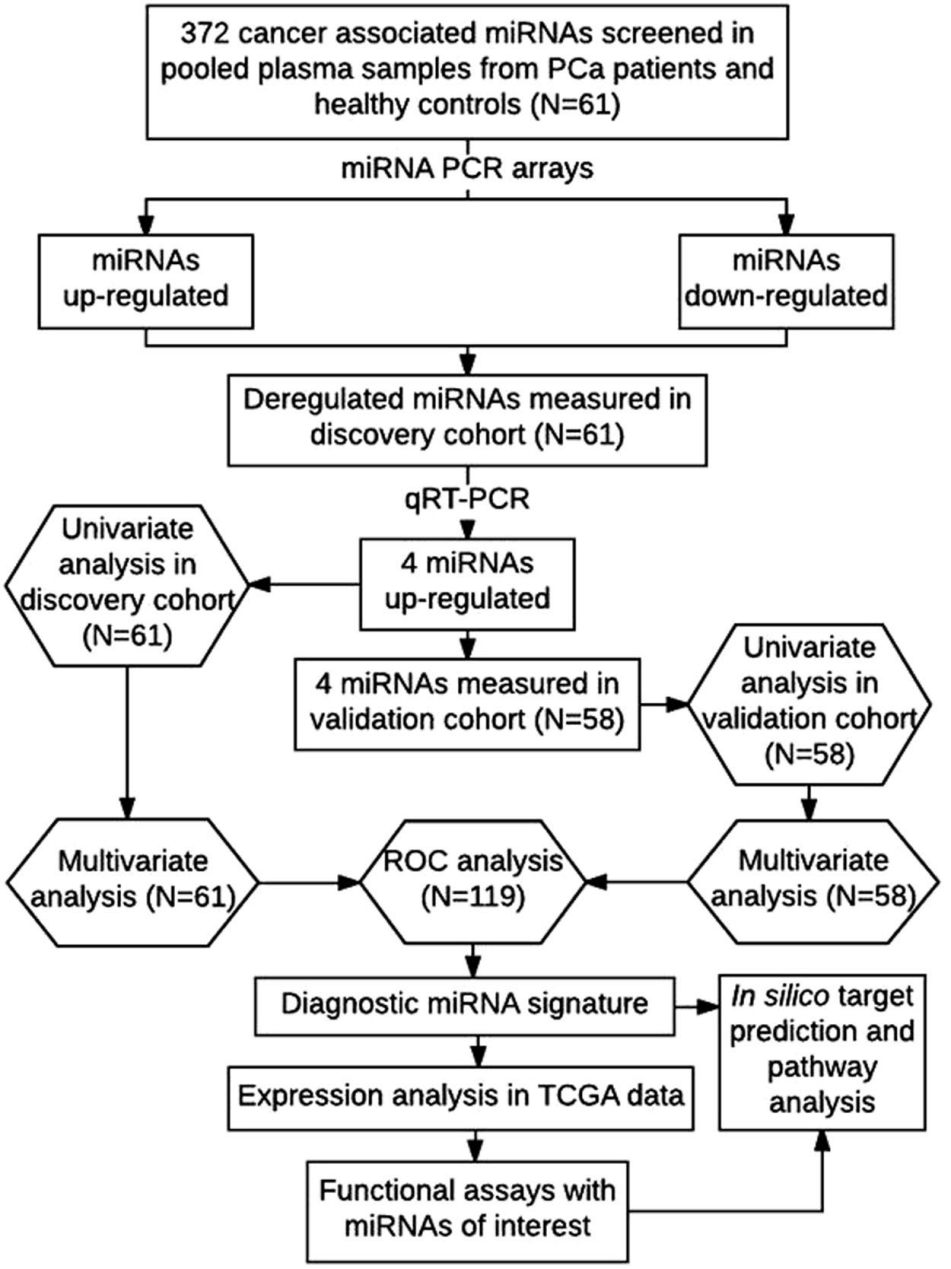
Study design and discovery and validation cohort study profiles. Flow diagram summarising the methodology and statistical approach used to identify diagnostic miRNAs followed by *in silico* target identification and pathway analysis, TCGA data expression analysis and functional assays.

Prior to RNA isolation, plasma samples in the discovery and validation cohorts were centrifuged for 10 min at 16,000 × g at 4°C and all plasma samples were screened for haemolysis using a Nanodrop ND**-**1000. For miRNA screening (Fig. 7), eight random plasma pools (two pools per group) were generated before RNA extraction for the control group and three patient groups in the discovery cohort using a randomising web-based tool (https://www.miniwebtool.com/random-picker/). In this way 150 µl of plasma required per pool was generated by dividing the total volume required (i.e. 150 µl) by the number of samples per pool. For example, the control group consisting of 19 healthy males was divided into two plasma pools of 10 and 9 controls. Each of the 10 controls in pool-1 contributed 15 µl of plasma, while each of the 9 controls in pool-2 contributed 16.67 µl of plasma. Similarly the three patient groups consisting of 12, 16 and 14 samples were divided into 6 × 2, 8 × 2 and 7 × 2 samples/pool respectively, and each patient per group contributed 25 µl of plasma in pools-3 and 4, 18.75 µl of plasma is pools-5 and 6, and 21.43 µl of plasma in pools-7 and 8. Therefore, total RNA was extracted from 150 µl of pooled plasma from each of the two pools per patient/control group in the discovery cohort, and eluted in 14 µl of RNase-free water using the miRNeasy Serum/Plasma kit (Qiagen) following the manufacturer’s instructions. 0.5 µg of bacterial ribosomal RNA (Roche) was added to each sample for increased RNA recovery, and 3.5 µl of miRNeasy Serum/Plasma Spike-In Control i.e. *cel*-miR-39 (at 1.6 × 10^8^ copies/µl) (Qiagen) was added to each sample as an internal control for plasma miRNA expression profiling and normalisation of qRT-PCR data as previously described^19^. 5 µl of extracted RNA from pooled plasma samples was reverse transcribed using the miScript II RT kit with HiSpec Buffer (Qiagen) according to the manufacturer’s instructions (Fig. 7).

### miRNA PCR array analysis

The resultant 20 µl cDNA, diluted in 90 µl of RNase-free water, was applied to the 384-well miScript miRNA PCR array (Qiagen) containing forward primers for the detection of 372 cancer-associated miRNAs and duplicates of 6 internal controls (Fig. 7). The qRT-PCR was run on a ViiA7 system (Applied Biosystems) and the data analysed using the GeneGlobe data analysis software (Qiagen). The *cel*-miR-39 Spike-In control was used by the program to calibrate the data sets, and the miRNA PCR array data was normalised by the global mean normalisation method^75^. This method automatically calculated a global C_T_ mean for the miRNA targets that were commonly expressed in all the samples being analysed after an initial calibration to the exogenous *cel*-miR-39 Spike-In control. For plasma miRNA expression profiling in individual samples, the *cel*-miR-39 Spike-In control was used as an internal control for normalisation of qRT-PCR data as previously described^19^. Fold regulation represents fold change values generated during miRNA expression profiling in a biologically meaningful way. The fold change was calculated using the equation 2^−∆∆CT^. For fold regulation, the fold change values less than 1 (meaning that the miRNA is down-regulated) was transformed by calculating the negative inverse. Therefore, the values remain the same while their representation is changed only. All C_T_ (number of cycles required for fluorescent signal to cross the set threshold; inversely proportional to miRNA levels) values >30 were considered as a negative call by the software. Therefore, the lower limit of detection was ≤30 C_T_ to avoid false positives. The final values of miRNA expression levels were generated in a biologically meaningful way as fold regulation i.e. the negative inverse of fold change. A 2.5 fold-regulation cut-off was used to shortlist deregulated miRNAs.

One of the challenges of miRNA profiling from plasma samples is the lack of established housekeeping genes for data normalisation. In general, miRNA profiling experiments use small noncoding RNAs, such as small nuclear or nucleolar RNAs (snRNAs/snoRNAs) for data normalisation^76^. However, they may not serve as ideal reference genes because snoRNAs and snRNAs do not share the same properties as miRNAs in terms of expression, transcription and processing^75^. Therefore, normalisation of data was performed using exogenous *cel*-miR-39 as an internal control and the global mean normalisation approach^16,19^.

### qRT-PCR validation

RNA extraction from individual plasma samples (N = 61) and cDNA synthesis were performed as described previously and the resultant 20 µl cDNA diluted 11x in 200 µl of RNase-free water. qRT-PCR of the shortlisted mature miRNAs was performed using miScript Primer Assays (forward primers) (Qiagen), Universal Primer (reverse primer) (Qiagen) and the QuantiTect SYBR Green PCR Master Mix (Qiagen) with 1 µl cDNA input for each 5 µl reaction in triplicates. The miRNA reverse transcription control (miRTC) primer assay (Qiagen) was used as an internal control to determine the reverse transcription efficiency for each sample. The qRT-PCR data was analysed using the externally spiked-in *cel*-miR-39 (Qiagen) as the housekeeping gene^19^ with a 2 fold-regulation cut-off used to identify deregulated miRNAs. The results obtained from the discovery cohort were further validated in the validation cohort (N = 58) using the same method as described above.

### Statistical analyses

Unpaired Mann-Whitney U tests were performed to identify differences in miRNA expression levels between patients and healthy controls in cohorts 1 and 2. The *p*-values were adjusted for multiple testing using Bonferroni correction. Univariate logistic regression was performed to determine the ability of individual miRNAs to predict disease occurrence in prostate cancer patients. The RNA concentration was set as an offset variable to account for the differences in the starting amounts of RNA. The capacity of combined miRNAs to distinguish between patients and healthy controls was evaluated by ROC curve analysis in the discovery (N = 61), validation (N = 58) and combined (N = 119) cohorts to increase the predictive power of the analysis. For ROC analysis of the miRNA panel, binary logistic regression analysis was performed for all four miRNAs at the same time enabling calculation of predicted probabilities of the miRNA combination which was further used to generate AUC values for the miRNA panel. The AUC is an effective and combined measure of sensitivity and specificity that describes the inherent validity of diagnostic tests. The ROC curve corresponding to progressively greater discriminant capacity of diagnostic tests is located closer to the upper left-hand corner away from the diagonal reference line^77^. A *p*-value of ≤0.05 was considered to be significant for all analyses. All statistical analyses were done using GraphPad Prism 7.02, SPSS and RStudio in consultation with qualified statisticians from the Queensland Facility for Advanced Bioinformatics (QFAB, UQ) and the Research Methods Group (RMG, QUT). An overall summary of the methodology is presented in Fig. 7.

### TCGA data and cell line expression analysis

Prostatic expression of the shortlisted miRNAs was assessed in a publicly available cohort comprising of 52 tumour and 52 non-malignant prostate tissues from The Cancer Genome Atlas (TCGA). The expression of the significantly deregulated miRNAs in prostate tumour tissues was evaluated in a panel of prostate cancer cell lines by qRT-PCR. RNA was extracted from cell lysates using the miRNeasy Micro kit (Qiagen) according to the manufacturer’s instructions followed by cDNA synthesis using the miScript II RT kit with HiSpec Buffer (Qiagen). qRT-PCR using the miScript primer assays (Qiagen) and QuantiTect SYBR Green PCR Master Mix (Qiagen) was performed as described previously.

### Cell growth and transfection

The LNCaP and PC3 prostate cancer cell lines were obtained from the American Type Culture Collection (ATCC, Rockville, MD, USA) and cultured in RPMI-1640 (Gibco) supplemented with 5% Fetal Bovine Serum (FBS) (Life Technologies). Cells were maintained in a humidified incubator (5% CO_2_ and 95% O_2_) at 37 °C and routinely tested for mycoplasma. For miRNA overexpression studies, LNCaP (15,000 cells/well) and PC3 (10,000 cells/well) were plated in a 96-well plate and transiently transfected with 5 nM and 20 nM of the respective miRVana miRNA Mimics (Life Technologies) of the shortlisted miRNAs in complex with Lipofectamine RNAiMAX and Lipofectamine 2000 Reagent (Life Technologies) in a 1:1 ratio according to the manufacturer’s instructions. The miRVana miRNA Mimic Negative Control #1 (Life Technologies) was used as the non-targeting control.

### Cell proliferation and migration assays

For cell proliferation assays 15,000 LNCaP and 10,000 PC3 cells were plated per well in a 96 well plate and allowed to grow for 4 hours before transient transfection with miRNA complexes as described above. For cell migration assays 96-well ImageLock plates (Essen BioScience) were coated with 40 μl Poly-L-ornithine (Sigma) overnight. 50,000 cells/well were plated the next day and allowed to form a confluent monolayer for 24 hours. The cells were treated with 10 µg/ml Aphidicolin (Sigma) (an antimitotic reagent) for 1 hour to minimise the effect of cell proliferation on migration. Wounds were created using a 96-pin WoundMaker (Essen BioScience) and the growth media was aspirated to remove cell debris. The cells were treated with 50 µl of miRNA complexes per well for half an hour before replenishment with 150 µl of RPMI containing 5% FBS. Percentage confluence (for proliferation) and relative wound density (for migration) was measured every 2 hours for 72 hours using the automated IncuCyte live cell imaging system (Essen BioScience) following the manufacturer’s protocol. All treatments were performed with six replicates in three independent experiments. Data analysis was performed using the IncuCyte software.

### Pathway Analysis

*In silico* network-based visual analysis was performed using the miRNet web-based platform to identify target genes and pathways potentially altered by the miRNA signature^21^. Forward mapping, which allows users to map from miRNAs to their targets, was performed by uploading the list of miRNA IDs of interest. After data processing, the results were presented as an interaction table with each row corresponding to one miRNA and its target. The table also provided hyperlinks to the corresponding databases from where the miRNA-target interactions were derived, together with references to PubMed literature. Network visualisation was performed through functional annotations based on the Kyoto Encyclopedia of Genes and Genomes (KEGG) pathway database, and the hypergeometric algorithm was used for enrichment analysis of input data to generate a list of miRNA target genes. This was further used as an input for in depth analysis using IPA (Qiagen) to determine top canonical pathways, upstream regulators and molecular and cellular functions of the miRNA target genes. The association of the deregulated target genes with disease phenotype was also confirmed using the Gene Set Enrichment Analysis (GSEA) computational method. The differential expression of the target genes in normal (N = 23) vs prostate carcinoma (N = 65) tissues were determined using the Oncomine cancer microarray database and integrated data-mining platform^68^. A *p*-value of ≤0.05 was considered to be significant for all the analyses.

### Validation of *in silico* target genes of miR-152-3p

LNCaP (300,000 cells/well) were plated in a 6 well plate for treatment with miR-152-3p and negative control mimics for 72 hours as described above for the collection of cell lysates (Norgen). This was followed by RNA extraction (Norgen), cDNA synthesis (Life Technologies) and qRT-PCR (Life Technologies) to determine the relative fold expression of target genes of miR-152-3p using target-specific primers (Sigma). Data was normalised to the housekeeping gene RPL32 and further normalised relative to the non-targeting negative control to determine relative fold expression. The differences in target gene expression between the negative control and miR-152-3p treated cells were assessed using an Unpaired t test, N = 3 (*p ≤ 0.05, **p ≤ 0.01, ***p ≤ 0.001 and ****p ≤ 0.0001).

### Data availability statement

The datasets generated during and/or analysed during the current study are available from the corresponding author on reasonable request.

## Electronic supplementary material

Supplementary Figures and Tables

Dataset 1

Dataset 3

Dataset 4

Dataset 5

Dataset 6

Dataset 7

Dataset 8

Dataset 9

Supplementary Video 1

Supplementary Video 2

Supplementary Video 3

Supplementary Video 4
