# Supplementary material for: A Plasma Biomarker Panel of Four MicroRNAs for the Diagnosis of Prostate Cancer
Source: Sci Rep. 2018 Apr 27;8:6653. doi: 10.1038/s41598-018-24424-w (PMC5923293; doi:10.1038/s41598-018-24424-w)
Supplement: Supplementary file 1 — Supplementary Figures and Tables [file 41598_2018_24424_MOESM1_ESM.pdf]

Title Page

Title: A Plasma Biomarker Panel of Four MicroRNAs for the Diagnosis of Prostate Cancer

Authors: Farhana Matin, Varinder Jeet, Leire Moya, Luke A. Selth, Suzanne Chambers, Australian Prostate Cancer BioResource, Judith A. Clements, Jyotsna Batra*

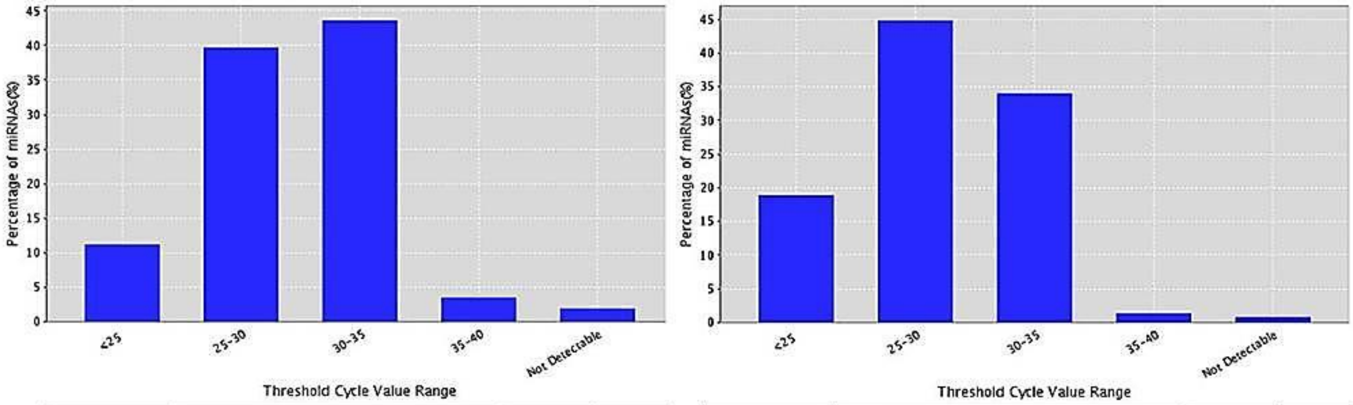

CT Range	Distribution of CT Values		Average	ST DEV
	Control pool-1	Control pool-2		
<25	39	47	43	5.66
25-30	158	147	152.5	7.78
30-35	162	173	167.5	7.78
Absent Calls	25	17	21	5.66
	Percent Distribution of CT Values			
<25	10.16%	12.24%	11.20%	0.01
25-30	41.15%	38.28%	39.71%	0.02
30-35	42.19%	45.05%	43.62%	0.02
Absent Calls	6.51%	4.43%	5.47%	0.01

CT Range	Distribution of CT Values		Average	ST DEV
	Patient pool-1	Patient pool-2		
<25	72	73	72.5	0.71
25-30	170	174	172	2.83
30-35	134	128	131	4.24
Absent Calls	8	9	8.5	0.71
	Percent Distribution of CT Values			
<25	18.84%	19.01%	18.92%	0.00
25-30	44.27%	45.40%	44.84%	0.01
30-35	34.89%	33.16%	34.03%	0.01
Absent Calls	1.99%	2.43%	2.21%	0.00

Supplementary Figure-S1. The distribution of miRNA C_T values between the healthy control and patient plasma pools during the screening study. In the control group, ~50% of the miRNAs had C_T values of <30, with 43.62% of miRNAs being in the 30-35 C_T range, whereas in the patient group, ~65% of the miRNAs had C_T values <30, with 44.84% of miRNAs being in the 25-30 C_T range. 5.47% miRNAs in the control group and 2.21% miRNAs in the patient group were undetected. The two patient pools are shown as an average of processed data from six pools. The raw unprocessed data from the miRNA PCR array is given in Supplementary Table-S1.

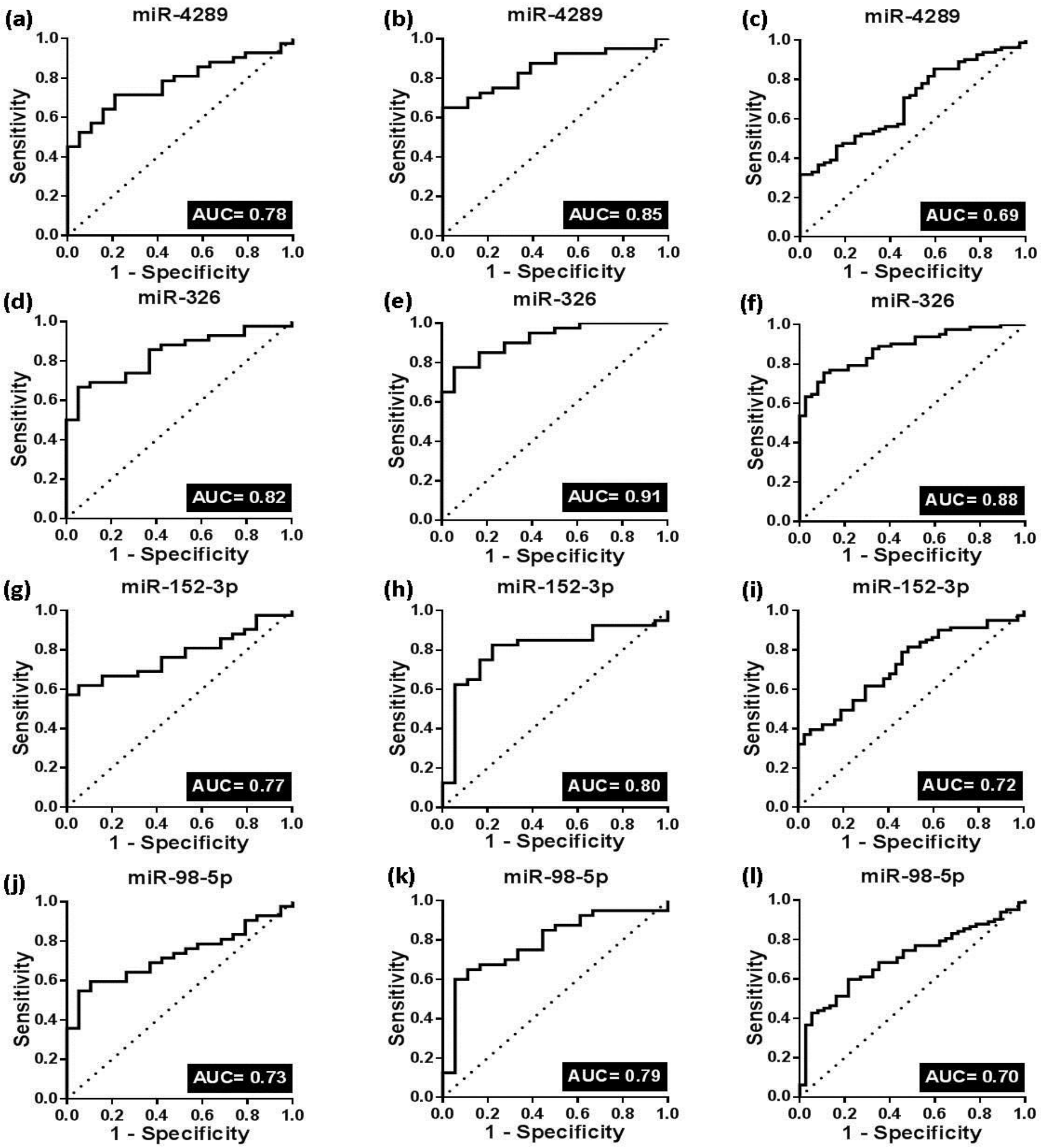

Supplementary Figure-S2. ROC curve analysis in the discovery (a, d, g, j), validation (b, e, h, k) and combined (c, f, i, l) cohorts comparing the ability of each miRNA in the panel to identify prostate cancer patients. A combined measure of the sensitivity and specificity of each miRNA in all three cohorts is represented by the Area under the curve AUC ($p \leq 0.05$). The diagonal reference line reflects the performance of the diagnostic test i.e. whether a test yields the positive or negative results by chance or due to a relation with the true disease status.

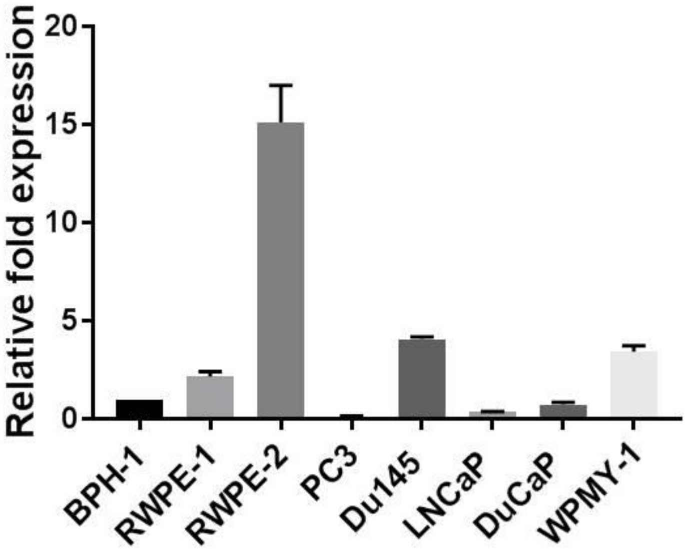

Supplementary Figure-S3. Endogenous expression of miR-152-3p in prostate cell lines. Data was normalised to the housekeeping gene RNU6 and further normalised relative to BPH-1 to determine fold change.

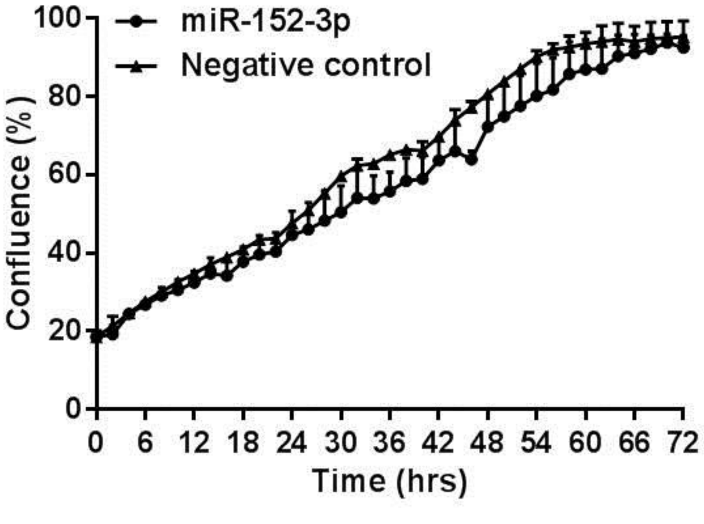

Supplementary Figure-S4. Overexpression of miR-152-3p using miRNA mimics in PC3 cells did not affect their proliferative capacity when compared with the negative control treated cells as measured as a change in percentage confluence by the IncuCyte live cell imaging system over a period of 72 hours.

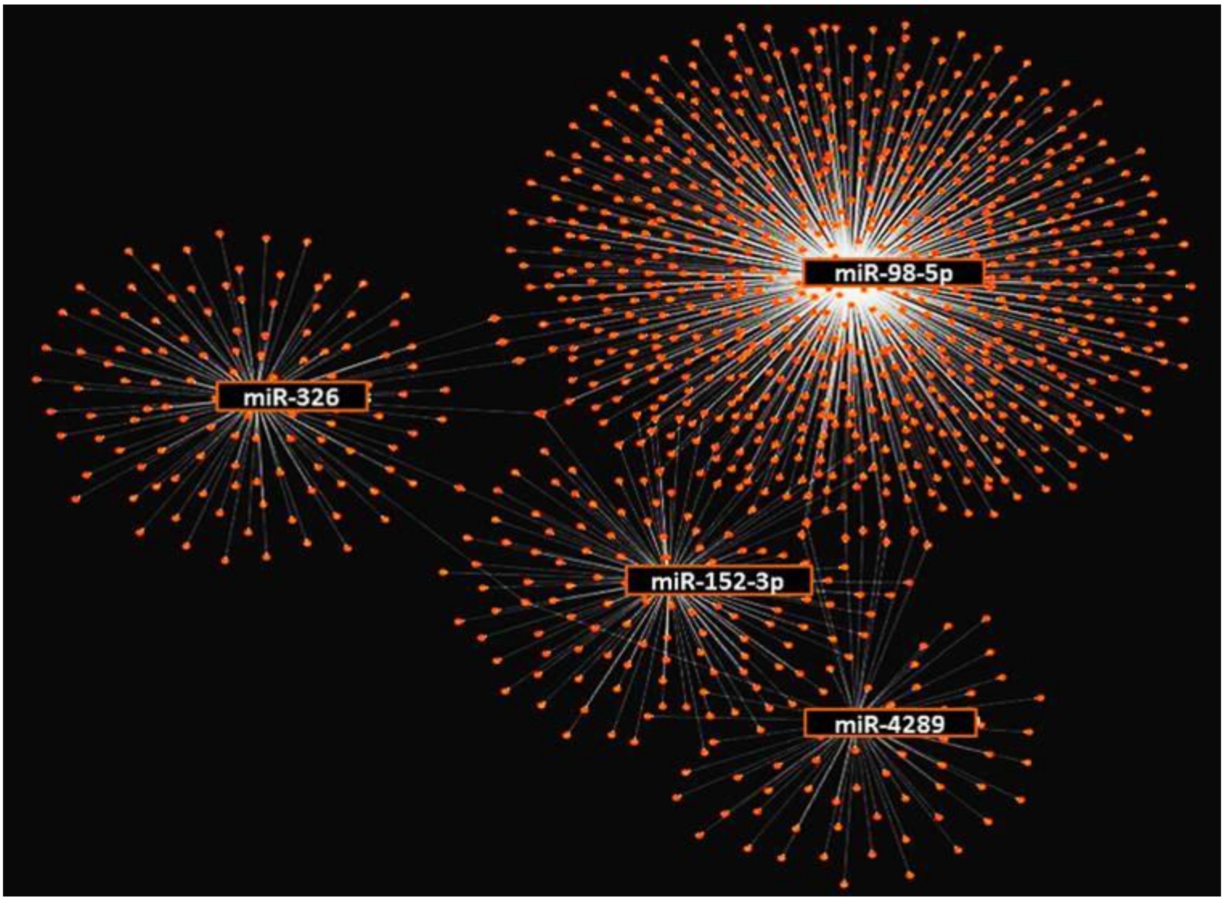

Supplementary Figure-S5. *In silico* pathway analysis using the miRNet platform generated a total of 261 candidate pathways and 1055 target genes (represented as round orange nodes) of the 4 miRNAs (default cut-off p≤ 1.00).

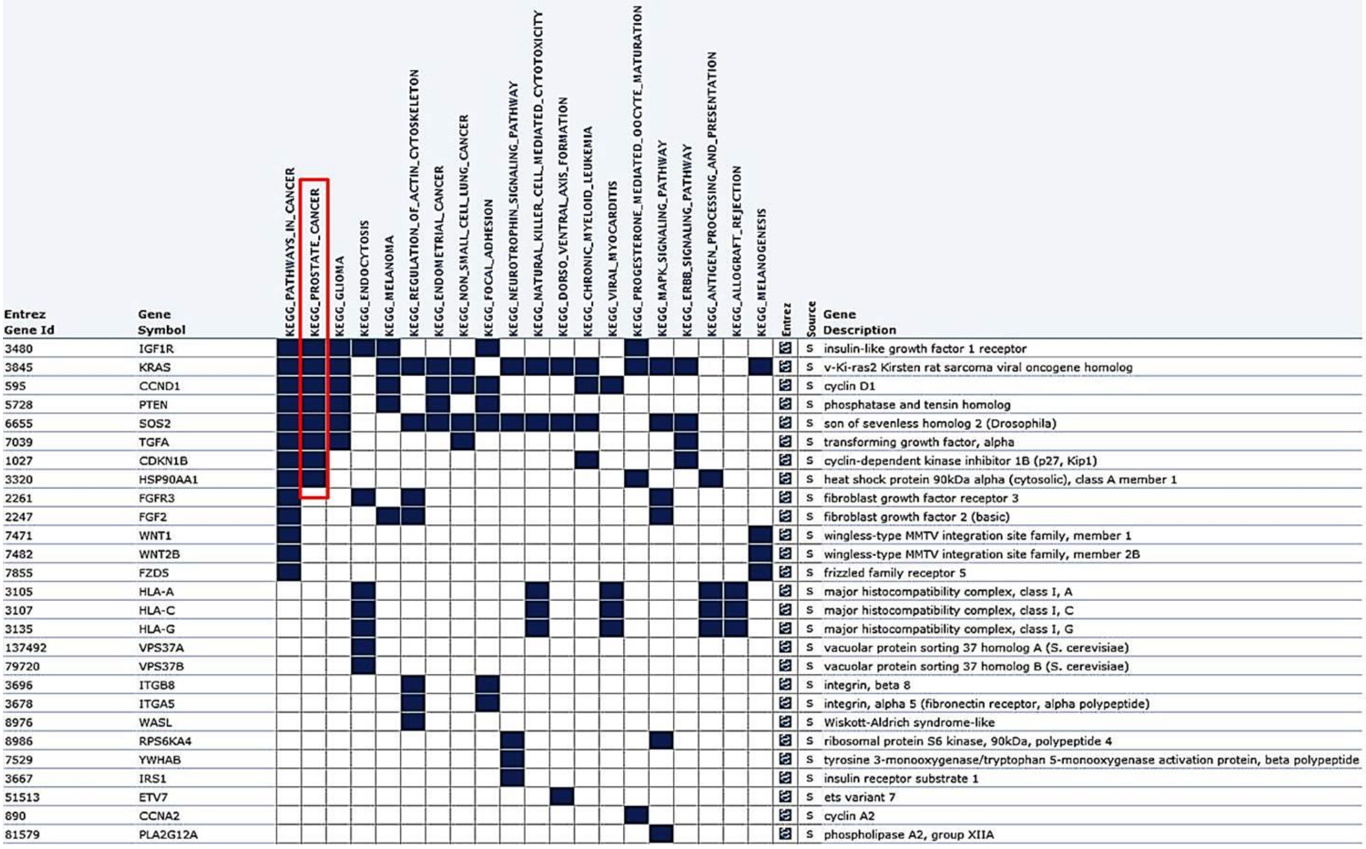

Supplementary Figure-S6. Of the eight target genes of miR-152-3p, six i.e. KRAS, CCND1, PTEN, SOS2, CDKN1B and HSP90AA1 (red rectangle) overlapped with GSEA where prostate cancer signalling was found to be the second most important pathway among the top 20 KEGG pathways.

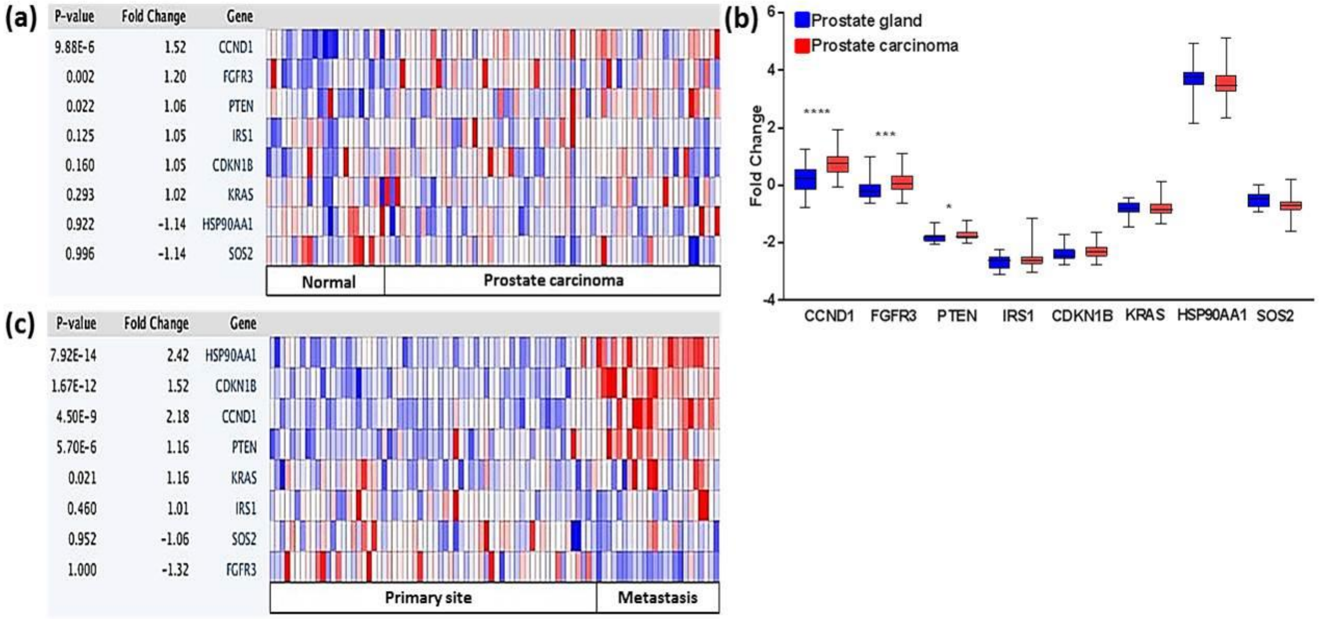

Supplementary Figure-S7. Oncomine data analysis of miR-152-3p targets to determine their expression in normal vs prostate carcinoma (a/b), and in primary vs metastatic cancer (c) in the Yu Prostate Cancer Dataset.

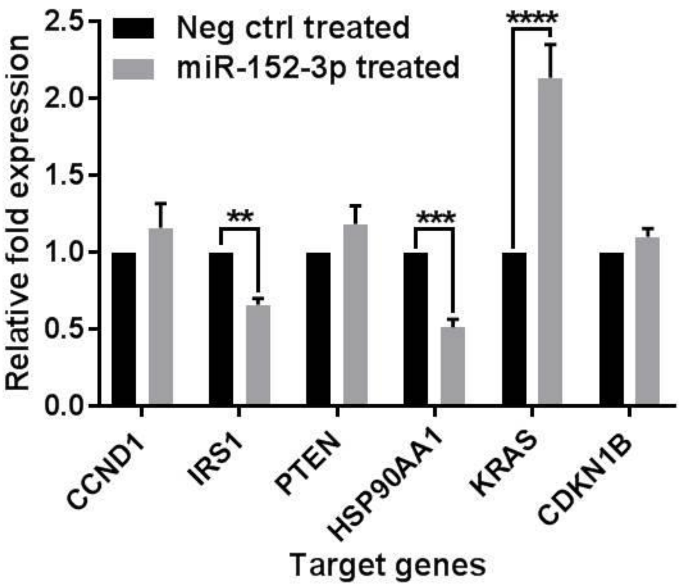

Supplementary Figure-S8. qRT-PCR analyses of six target genes of miR-152-3p in LNCaP cells after treatment with non-targeting negative control and miR-152-3p mimics for 72 hours. miR-152-3p induced a reduction in IRS1 ($p= 0.0014$) and HSP90AA1 ($p= 0.0007$) mRNA, and increased the expression of KRAS ($p< 0.0001$) in LNCaP cells. Data was normalised to the housekeeping gene RPL32 and further normalised relative to the non-targeting negative control to determine relative fold expression. The differences in target gene expression between the negative control and miR-152-3p treated cells were assessed using an Unpaired t test, N=3 ($p\leq 0.05$*, $p\leq 0.01$**, $p\leq 0.001$*** and $p\leq 0.0001$****).

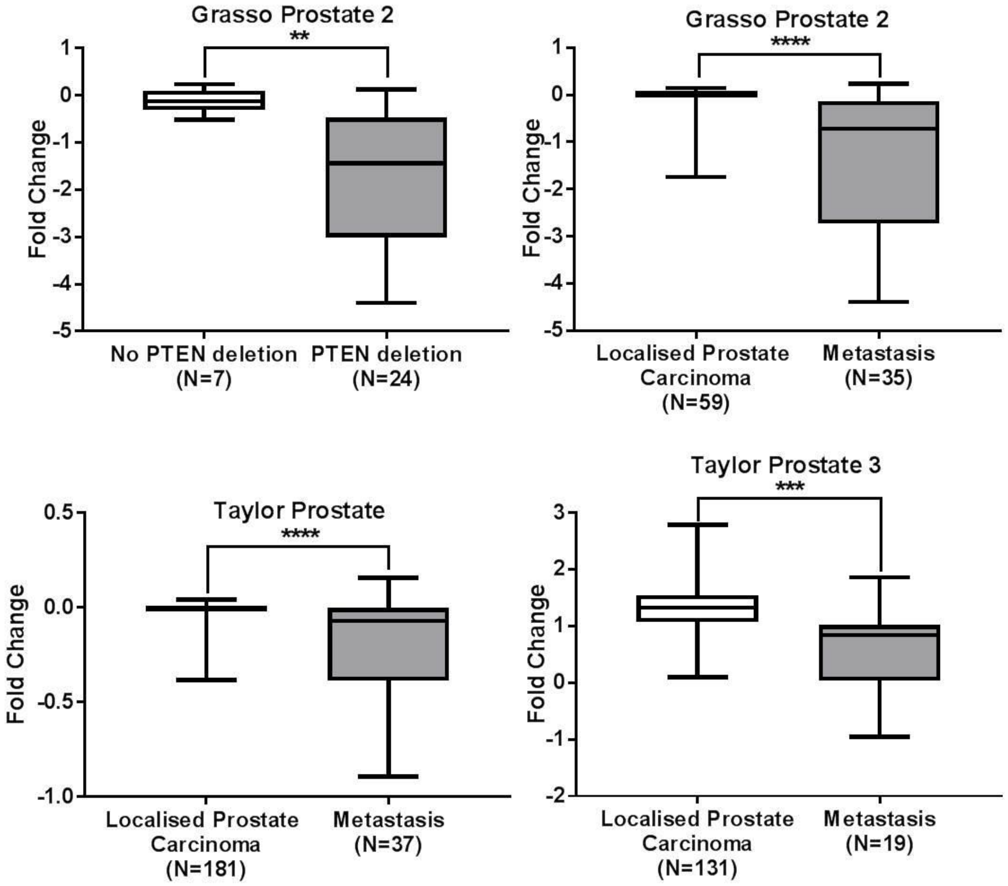

Supplementary Figure-S9. (a) Oncomine data analysis of PTEN copy number deletion in prostate carcinoma metastasis in the Grasso Prostate 2 Cancer Dataset ($p= 0.0011$). (b) Copy number deletion of PTEN in localised vs metastatic prostate carcinoma in the Grasso Prostate 2 Cancer Dataset ($p< 0.0001$). (c) Copy number deletion of PTEN in localised vs metastatic prostate carcinoma in the Taylor Prostate Cancer Dataset ($p< 0.0001$). (d) Expression of PTEN in localised vs metastatic prostate carcinoma in the Taylor Prostate 3 Cancer Dataset ($p= 0.0001$). Unpaired Mann-Whitney U tests were performed to identify differences in PTEN expression levels ($p\leq 0.05$*, $p\leq 0.01$**, $p\leq 0.001$*** and $p\leq 0.0001$****).

Supplementary Table-S1. <u>(Attached as an Excel file)</u> The expression level (Ct value) of each miRNA in the PCR-array during miRNA screening from pooled plasma samples.

Genes Over-Expressed in		
Position in PCR array	Mature ID	Fold Regulation
A06	hsa-miR-152-3p	2.8014
D21	hsa-miR-590-5p	3.3123
I01	hsa-miR-98-5p	2.9646
L24	hsa-miR-301a-3p	4.3104
M05	hsa-miR-4289	2.6138
M22	hsa-miR-1260b	2.7533
M24	hsa-miR-326	2.706
Genes Under-Expressed in		
Position in PCR array	Mature ID	Fold Regulation
B08	hsa-miR-4732-5p	-2.5856
K11	hsa-miR-671-3p	-2.7969
L07	hsa-miR-675-3p	-2.5589
M01	hsa-miR-181d-5p	-2.5826

Supplementary Table-S2. Of the 372 cancer-associated miRNAs, 11 deregulated miRNAs with a fold regulation cut off of 2.5 were shortlisted for validation in the discovery cohort.

Supplementary Table-S3. <u>(Attached as an Excel file)</u> The clinical characteristics of patients and healthy controls in the discovery and validation cohorts.

Supplementary Table-S4. <u>(Attached as an Excel file)</u> miRNA expression in prostate tumour (N=52) and adjacent non-malignant (N=52) tissues in the TCGA dataset.

Supplementary Table-S5. <u>(Attached as an Excel file)</u> The miRNet web-based platform generated a total 1055 target genes (default cut-off $p \leq 1.00$) of which 65 genes were targeted by miR-4289, 120 genes by miR-326, 131 genes by miR-152-3p and 739 genes by miR-98-5p.

Supplementary Table-S6. <u>(Attached as an Excel file)</u> The miRNet web-based platform generated a total of 261 candidate pathways (default cut-off $p \leq 1.00$). Of the 261 pathways, 25 pathways were significant ($p \leq 0.05$) with the prostate cancer pathway being eleventh and the viral carcinogenesis pathway being the topmost in the list.

Supplementary Table-S7. <u>(Attached as an Excel file)</u> Ingenuity pathway analysis (IPA) generated a total of 275 canonical pathways for the 131 miRNet predicted targets of miR-152-3p. Prostate cancer signalling was the sixth canonical pathway in the list and

corresponded to 8 deregulated target genes i.e. FGFR3, IRS1, SOS2, HSP90AA1, KRAS, CDKN1B, CCND1 and PTEN of miR-152-3p (-log *p*= 5.75).

Supplementary Table-S8. **(Attached as an Excel file)** Analysis of molecular function of miR-152-3p target genes confirmed their involvement in cellular processes such as cell cycle, cell morphology, cell growth and proliferation and cell movement.

Supplementary Table-S9. **(Attached as an Excel file)** IPA generated 1055 upstream regulators of the eight target genes of miR-152-3p.
